# RE: Extending section 12 approval under the Mental Health Act to professions other than medicine

**DOI:** 10.1192/bjb.2025.33

**Published:** 2025-06

**Authors:** Claire Hilton, Alan Cohen

**Affiliations:** 1 Former Psychiatrist, Now Historian, Centre for Interdisciplinary Research on Mental Health, Birkbeck, University of London, London, UK; 2 Retired GP

## MHA 1983 Section 12 approval for general practitioners

At the beginning of their editorial on the Mental Health Act (MHA) 1983 and section 12 approval, John Taylor and Carole Burrell indicate their surprise that general practitioners (GPs) may be approved under the section without specialist mental health training. Given the prominence of their statement, it is even more surprising that they make no further mention of GPs in their discussion or offer any explanation.

When the MHA 1983 came into force, general practice was very different from what it is today. In particular, it was based on long-term relationships between patients and practitioners. This relational model was disrupted in the early 1990s with the purchaser–provider split, which introduced financial competition to improve efficiency. After that, the dominance of guidelines and pathways further damaged the relational model.^1^ Today, one might describe another phase of development: the austerity, post-Covid era, with increased demand on GPs, more task-based and bureaucratic dimensions, and greater use of online and telephone consultations, plus challenges in maintaining the workforce. The Royal College of General Practitioners reported that in 2024 ‘63% of GP members do not feel they have enough time during appointments to build the patient relationships they need to deliver care’.^2^ All the changes, but especially the decline of the relational model, have had ramifications for patients suffering from mental problems, arguably more so than for those with other sorts of disorder.

Given the historical context, it was unsurprising that in 1983, GPs were in the privileged position of having special status regarding experience and approval under the MHA, without formal postgraduate psychiatric training. They had specialist knowledge because of their long-term relationships with their patients, knowing about the nature and course of their mental and physical illnesses, their needs, their families and outcomes of earlier treatment. GPs frequently also had long-term working relationships with the mental health teams in their local catchment area. Through these patient and secondary care relationships, GPs accumulated person-centred expert knowledge.

Taylor and Burrell also noted that Sir Simon Wessely’s Independent Review of the MHA^3^ did not address section 12 approval. Regarding the anomaly of section-12-approved GPs, given that the report preceded the massive changes in primary care since Covid, it is less surprising that this was not one of his concerns. Re-establishing the relational model of general practice, together with improved resourcing and closer collaboration between primary and secondary care services,^4^ would be preferable to changing the law in this regard.

## References

[1] Cohen A, Tylee A, Thurston L, Hall J. *Witness Seminar: History of Primary Care Mental Health in England 1948-2019*. Royal College of Psychiatrists, 2022 (https://www.rcpsych.ac.uk/about-us/library-and-archives/archives/witness-seminars/witness-seminar--history-of--primary-care-mental-health-in-england-1948-2019).

[2] Royal College of General Practitioners (RCGP). *Key General Practice Statistics for England*. RCGP, 2025 (https://www.rcgp.org.uk/representing-you/key-statistics-insights).

[3] Department of Health and Social Care. *Independent Review of the Mental Health Act*. Department of Health and Social Care, 2018 (https://www.gov.uk/government/groups/independent-review-of-the-mental-health-act).

[4] British Medical Association (BMA). ‘It’s Broken’: Doctors’ Experiences on the Frontline of a Failing Mental Healthcare System. BMA, 2024 (https://www.bma.org.uk/media/ddclsiii/bma-mental-health-report-2024-web-final.pdf).

